# A Southeast Asian Experience in Biodegradable Implants: Tips and Tricks for Radiological Findings

**DOI:** 10.7759/cureus.95871

**Published:** 2025-11-01

**Authors:** Teddy Cheong, Karen Fernandes, Joe Francis, Le Roy Chong, Charles Kon

**Affiliations:** 1 Orthopedic Surgery, Changi General Hospital, Singapore, SGP; 2 Diagnostic Radiology, Changi General Hospital, Singapore, SGP

**Keywords:** bioabsorbable screws, biodegradable implants, foot and ankle surgery, magnesium-based implants, musculoskeletal radiology

## Abstract

Nondegradable implants, such as titanium or steel screws, are commonly used in orthopedic surgery as they provide strength and stability. However, metallic implants have been associated with multiple issues, such as additional surgery to remove hardware, interference with imaging modalities, and stress shielding. Biodegradable magnesium-based implants are an innovative alternative due to good biocompatibility and osteoconductivity, similar to Young’s elastic modulus to bone, and they exhibit less metallic distortion in imaging modalities. The MAGNEZIX compression screw became the first magnesium implant to be approved for use in humans and has been widely used ever since, most commonly in foot and ankle conditions, with generally good outcomes. Despite its advantages, implant biodegradation introduces unique imaging challenges that can be misinterpreted as complications. This article aimed to familiarize radiologists and clinicians with the expected imaging findings of magnesium-based implants to reduce unnecessary advanced imaging and misdiagnosis. Pre-operative and post-operative radiological images of 75 patients with foot and ankle injuries who were treated with MAGNEZIX screws between 2020 and 2022 in Changi General Hospital were retrospectively evaluated. Selected cases are presented in detail to illustrate key principles in understanding radiological findings of MAGNEZIX screws.

Seven cases are presented in detail with pre-operative and serial post-operative images, along with each case emphasizing a key learning point essential to understanding when interpreting radiological images of patients who receive MAGNEZIX screws as part of their treatment. Key takeaways include the following points: (1) recognizing the normal evolution and degradation pattern of magnesium implants and being aware of potential pitfalls, (2) the importance of correlating radiological findings with clinical assessment, (3) effective multidisciplinary communication, and (4) staying updated with ongoing literature on degradable magnesium-based implants. Biodegradable implants, such as MAGNEZIX screws, are an innovative and effective alternative to metallic implants in orthopedic surgery. However, knowledge of the expected imaging findings of magnesium-based implants after insertion is essential in reducing unnecessary advanced imaging and misdiagnosis.

## Introduction

In orthopedic surgery, a biomechanically robust implant is required to achieve stable fixation of the fracture. Nondegradable implants, such as titanium or steel screws, are commonly used as they provide strength and stability. However, metallic implants have been associated with multiple disadvantages. This includes implant-related complications, additional surgery to remove hardware, interference with imaging modalities, and stress shielding [1-4]. To overcome these issues, biodegradable implants have been developed and used over the years. Polymer-based implants were the first to be used. However, these were mechanically weaker than metallic implants [5]. They were also associated with foreign body reactions and osteolysis, as degradation occurred via hydrolysis, which led to acidity and inflammatory changes [6,7].

Biodegradable magnesium-based implants are an innovative alternative. In 2013, MAGNEZIX compression screw (Hanover, Germany: Syntellix AG) was the first magnesium implant to be approved for humans. It is an aluminum-free magnesium alloy that is classified as an MgYREZr alloy. Studies have shown that these implants have good biocompatibility and osteoconductivity without acute, subacute, or chronic toxicity [8]. Furthermore, they have a similar Young's elastic modulus to natural bone with higher stability compared to polymers [9]. They also have less metallic distortion and interference in imaging modalities when compared to metal [10].

MAGNEZIX screws have been mostly used in foot and ankle conditions, with multiple studies reporting good outcomes and safety profile that is comparable to nondegradable [11-17]. It has also been used with good effect in fractures involving other joints, such as the elbow [18,19]. Despite the significant advantages of MAGNEZIX screws, implant biodegradation (through surface oxidation, corrosion, and finally dissolution) introduces unique imaging challenges that can be misinterpreted as complications [20]. This article aimed to familiarize radiologists and clinicians with the expected imaging findings of magnesium-based implants to reduce unnecessary advanced imaging and misdiagnosis.

## Materials and methods

This was a retrospective, single-center study of patients who received MAGNEZIX screws fixation for foot and ankle injuries between 2020 and 2022. Inclusion criteria are as follows: all foot and ankle injuries that received MAGNEZIX screw fixation, regardless of the number of screws used, and whether it was used alone or in conjunction with other fixation devices. Exclusion criteria are as follows: all foot and ankle injuries that were not treated with MAGNEZIX screws as part of fixation. Pre- and post-operative radiological images from 75 patients were evaluated to assess the radiographic features of MAGNEZIX screws. Seven selected cases are presented in detail to illustrate key principles in understanding radiological findings of MAGNEZIX screws. The SingHealth Centralized Institutional Review Board approved this study.

## Results

Seven cases were selected to be presented in detail in this article to illustrate principles of radiological findings concerning MAGNEZIX screws. Each case highlights a key learning point crucial for interpreting radiological images of patients who receive MAGNEZIX screws as part of their treatment (Table 1).

**Table 1:** Summary of the included cases.

Case number	Case title	Case highlights
Case 1	Long-term temporal evolution and degradation of magnesium implants	To understand the expected long-term imaging appearance of implant degradation, which could be mistaken for pathology, to avoid unnecessary advanced imaging
		Illustrate that magnesium implants produce fewer imaging artifacts than metallic implants
Case 2	Uneventful healing and gradual resorption of magnesium implants	An uneventful post-operative healing case that demonstrates the typical bioabsorption and healing process seen in magnesium-based implants
Case 3	Differentiating normal magnesium implant degradation from hardware failure	This case highlights the importance of distinguishing expected material fragmentation and degradation from true hardware failure
Case 4	Recognizing benign peri-implant lucencies in magnesium implants	Case example illustrating the importance of not misinterpreting persistent peri-implant radiolucent zones as signs of infection or loosening
Case 5	Exaggerated yet benign peri-implant radiolucencies	This case demonstrates that even substantial peri-implant gas can be an expected and benign finding with magnesium implants
Case 6	Red flags in peri-implant lucencies - when to suspect pathology	Complication: infection
		This case illustrates the principle that persistent or progressive lucency, bone resorption, or soft tissue swelling should raise suspicion and require careful clinical correlation
Case 7	Pathological peri-implant lucencies with hardware loosening and infection	Complication: infection and hardware failure
		Case example that underscores the importance of distinguishing expected peri-implant gas from progressive lucencies and structural changes, as clinical deterioration in such contexts may indicate true hardware failure and warrant surgical intervention

Case 1: long-term temporal evolution and degradation of magnesium implants

**Figure 1:**
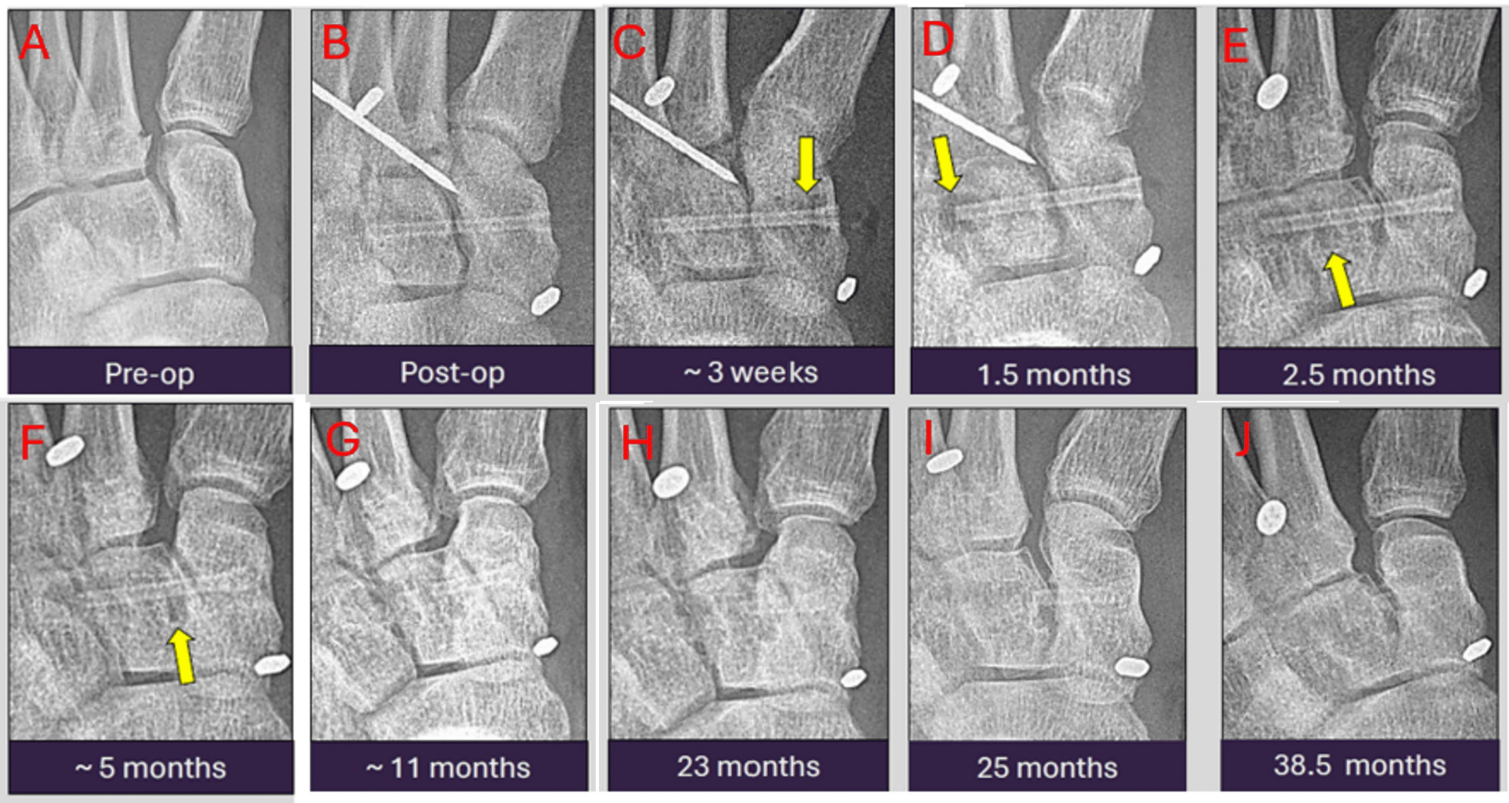
Serial antero-posterior radiographic images from pre-operation to 38 months post-operation showing early peri-implant radiolucency (arrows) which represent hydrogen gas formation, while gradual implant degradation with cortical remodeling reflect normal bioabsorption. The radiographs were taken at the following time points: pre-operation (A), immediately post-operation (B), three weeks (C), 1.5 months (D), 2.5 months (E), five months (F), 11 months (G), 23 months (H), 25 months (I), and 38.5 months (J) post-operation.

**Figure 2:**
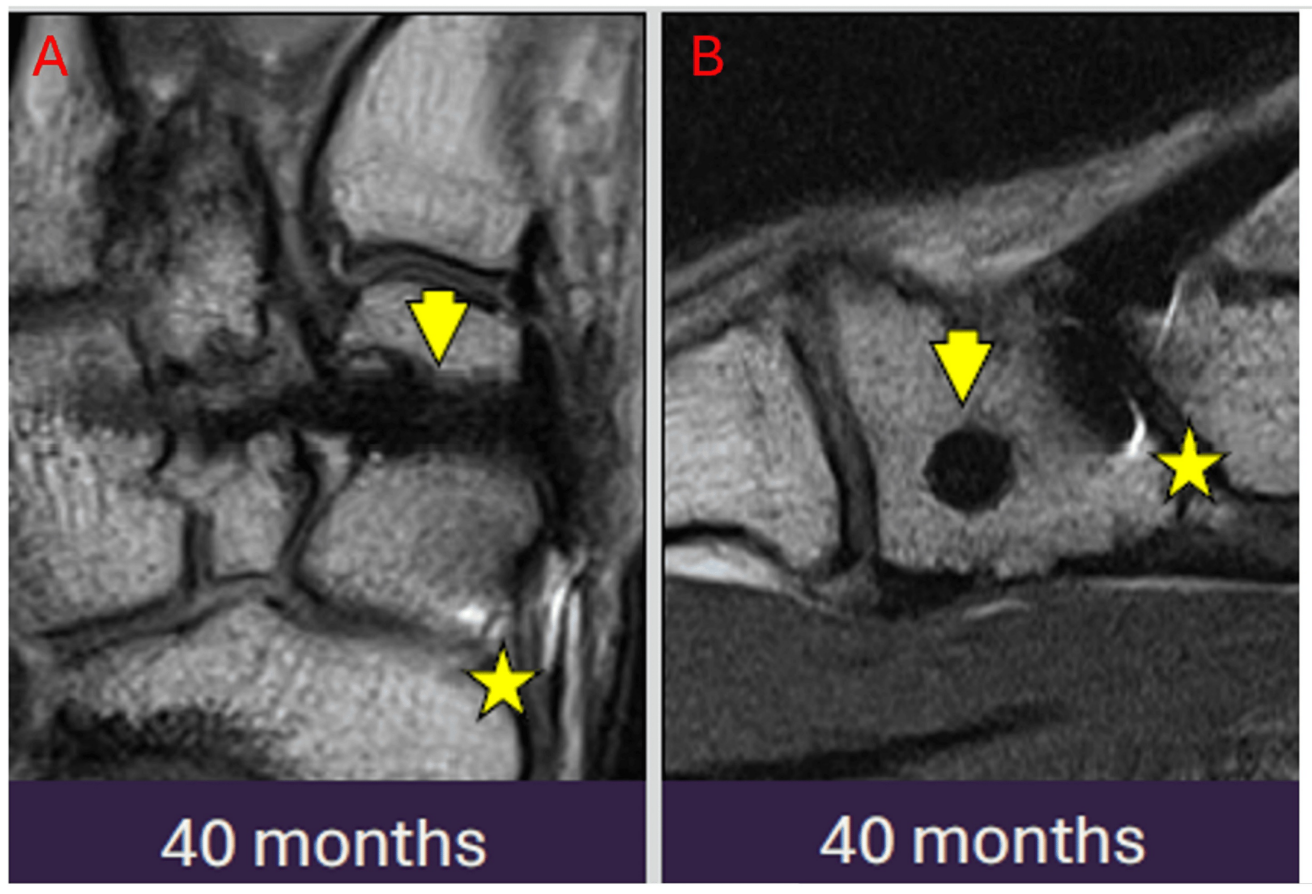
Forty months post-operative magnetic resonance imaging showing more artifacts from the titanium implant (star) as compared to the remnants of the magnesium implant which appear hypointense (arrowhead). The images show an axial cut (A) and a sagittal cut (B).

This case illustrates the expected long-term temporal evolution and fracture healing in a 27-year-old patient with a Lisfranc injury and multiple metatarsal fractures. Fixation was achieved using a 3.2 mm MAGNEZIX screw, Mini TightRope, and Kirschner wire. Serial radiographs spanning from October 2020 to February 2023 are shown. Early peri-implant radiolucency represents hydrogen gas formation, while gradual implant degradation with cortical remodeling reflects normal bioabsorption. These changes may mimic implant loosening, fracture, or infection if not correctly recognized (Figures 1A-1J). Despite apparent fragmentation over time, magnetic resonance imaging (MRI) at 40 months post-operation confirms complete fracture healing with no mechanical failure or complications. This case serves as an example for radiologists and clinicians to understand the expected long-term imaging appearance of magnesium implant degradation, which can otherwise be mistaken for pathology. We also noted that on the MRI, the titanium implants show markedly more artifacts than the remnants of the magnesium implant, which appear hypointense (Figures 2A, 2B).

Case 2: uneventful healing and gradual resorption of magnesium implants

**Figure 3:**
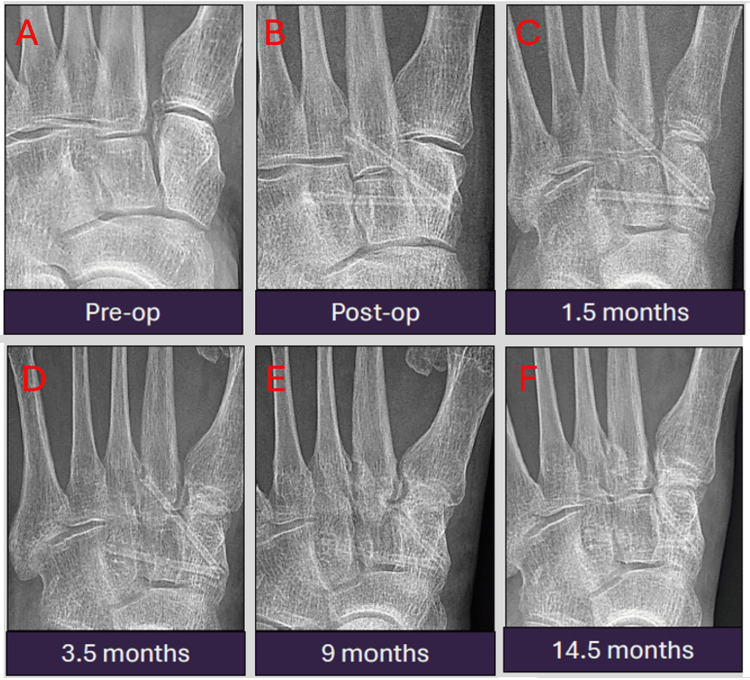
Serial antero-posterior radiographic images from pre-operation to 14.5 months post-operation showing stable implant position and progressive reduction in implant density and size, reflecting the bioabsorption process. The radiographs were obtained at pre-operation (A), immediately post-operation (B), 1.5 months (C), 3.5 months (D), nine months (E), and 14.5 months (F) post-operation.

This is a case of a 68-year-old patient who sustained an eversion injury of the ankle and underwent inter-cuneiform fixation followed by Lisfranc fixation with two 2.7 mm MAGNEZIX screws. The radiographs, spanning from December 2022 to February 2024, demonstrate the expected uneventful healing of the fracture alongside the gradual resorption of the magnesium implants. Mild peri-implant lucencies are visible, representing hydrogen gas release. Notably, there is a stable implant position and progressive reduction in implant density and size, reflecting the bioabsorption process (Figures 3A-3F). This case exemplifies the typical post-operative bony healing process, resembling what is expected with conventional metallic implants.

Case 3: differentiating normal magnesium implant degradation from hardware failure

**Figure 4:**
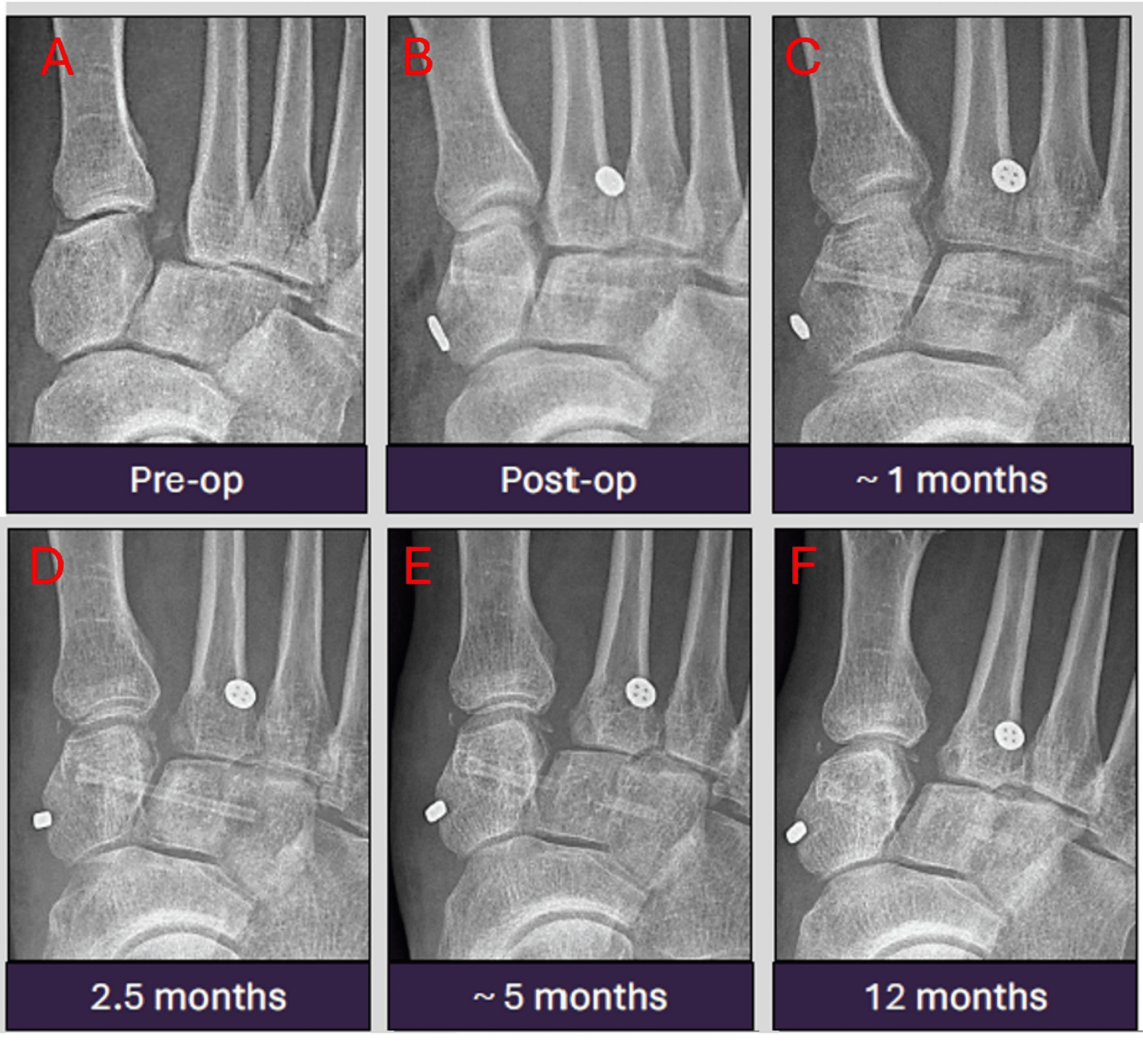
Serial antero-posterior radiographic images from pre-operation to 12 months post-operation showing expected progressive implant fragmentation and degradation without migration or loss of reduction. The radiographs were taken at pre-operation (A), immediately post-operation (B), one month (C), 2.5 months (D), five months (E), and 12 months (F) post-operation.

This is a case of a 48-year-old patient who sustained a Lisfranc injury, treated with inter-cuneiform fixation using a 3.2 mm MAGNEZIX screw, followed by Mini TightRope fixation. The serial radiographs demonstrate the normal temporal evolution of a magnesium implant. Notably, there is progressive implant fragmentation and degradation, i.e., “breaks” and “blurs,” most pronounced at ~5 months and 12 months. Despite appearances, there is no implant migration or loss of reduction (Figures 4A-4F). The post-operative recovery period was uneventful. This case highlights a common pitfall in interpreting magnesium screw degradation - mistaking expected material breakdown for hardware failure.

Case 4: recognizing benign peri-implant lucencies in magnesium implants

**Figure 5:**
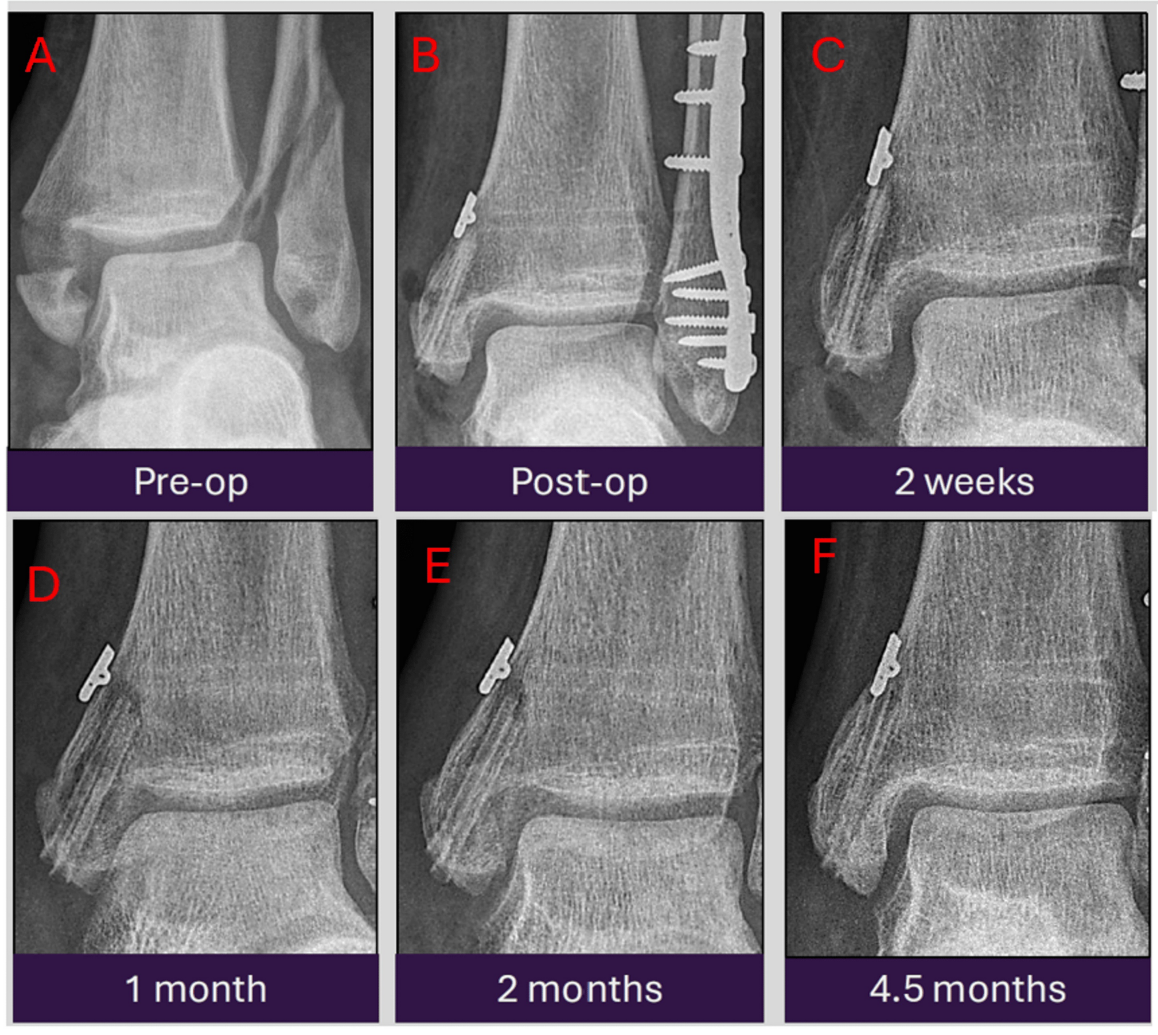
Serial antero-posterior radiographic images from pre-operation to 4.5 months post-operation showing persistent peri-implant radiolucencies which represent hydrogen gas released during implant biocorrosion, a benign and expected finding. The radiographs were obtained at pre-operation (A), immediately post-operation (B), two weeks (C), one month (D), two months (E), and 4.5 months (F) post-operation.

A 22-year-old patient sustained a bimalleolar Weber C ankle fracture and was treated with surgical fixation using a Zimmer plate, ZipTight, and MAGNEZIX screws. Serial radiographs demonstrate persistent peri-implant radiolucent zones. The recovery period was uneventful. These "bubbles" represent hydrogen gas released during the implant's biocorrosion, a benign and expected finding with magnesium implants (Figures 5A-5F). Recognizing this as benign gas formation is crucial to avoid misinterpreting it as infection or implant loosening, thus preventing unnecessary investigations or interventions.

Case 5: exaggerated yet benign peri-implant radiolucencies

**Figure 6:**
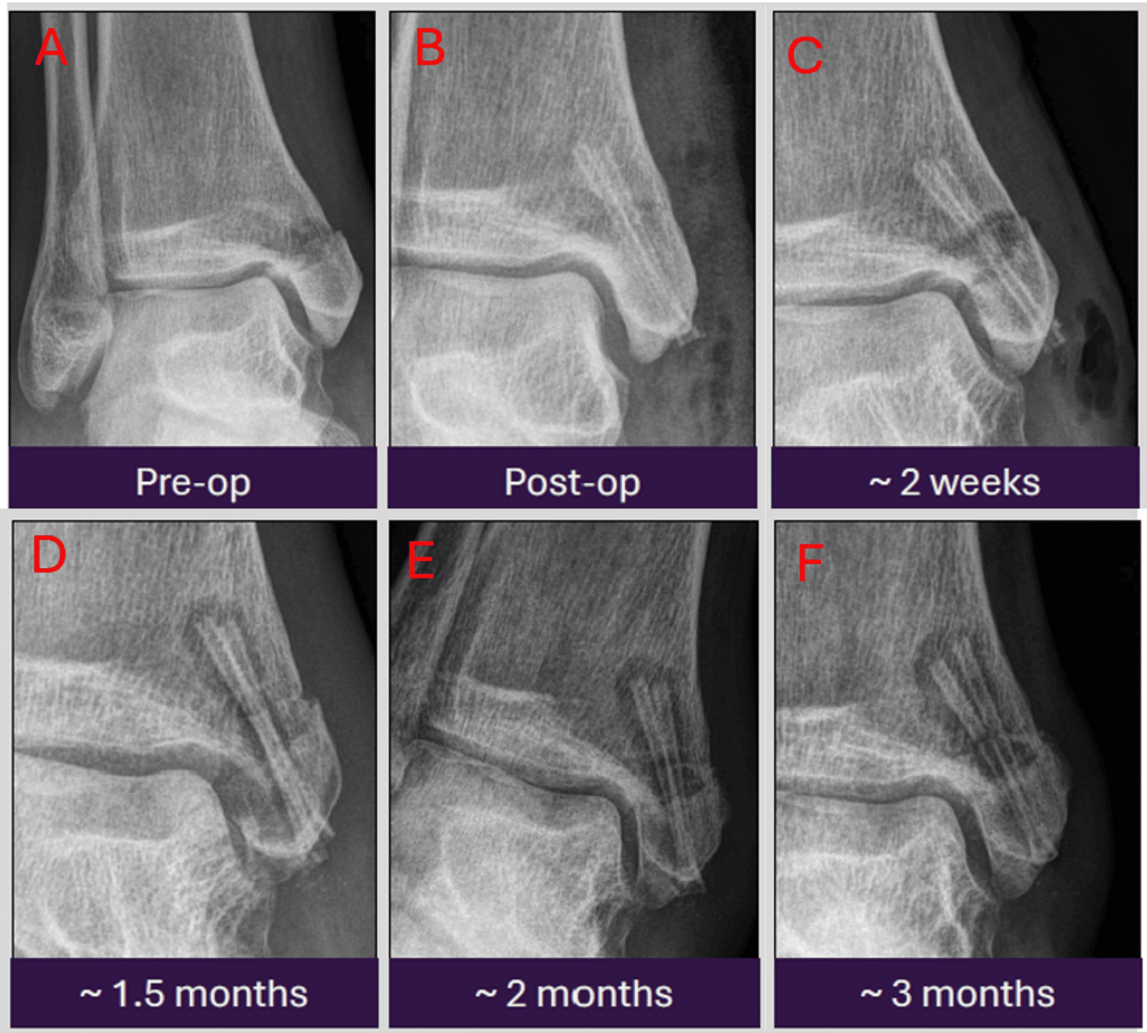
Serial antero-posterior radiographic images from pre-operation to three months post-operation showing persistent significant peri-implant radiolucencies which are considered a normal variant of the hydrogen gas release during biocorrosion. The radiographs were taken at pre-operation (A), immediately post-operation (B), two weeks (C), 1.5 months (D), two months (E), and three months (F) post-operation.

This example depicts a 54-year-old patient with a medial malleolar ankle fracture treated with MAGNEZIX screws. Radiographs demonstrate pronounced peri-implant lucencies and soft-tissue gas, particularly noticeable around two weeks and persisting up to three months post-operation. Despite their seemingly exaggerated appearance, these findings are considered a normal variant of the hydrogen gas release during the implant's biocorrosion. Fracture healing and patient recovery were uneventful (Figures 6A-6F). This case highlights that even significant peri-implant gas is an expected and benign phenomenon with magnesium implants, emphasizing the importance of recognizing the wide spectrum of normal degradation appearances and correlation with clinical findings to avoid misdiagnosis.

Case 6: red flags in peri-implant lucencies - when to suspect pathology

**Figure 7:**
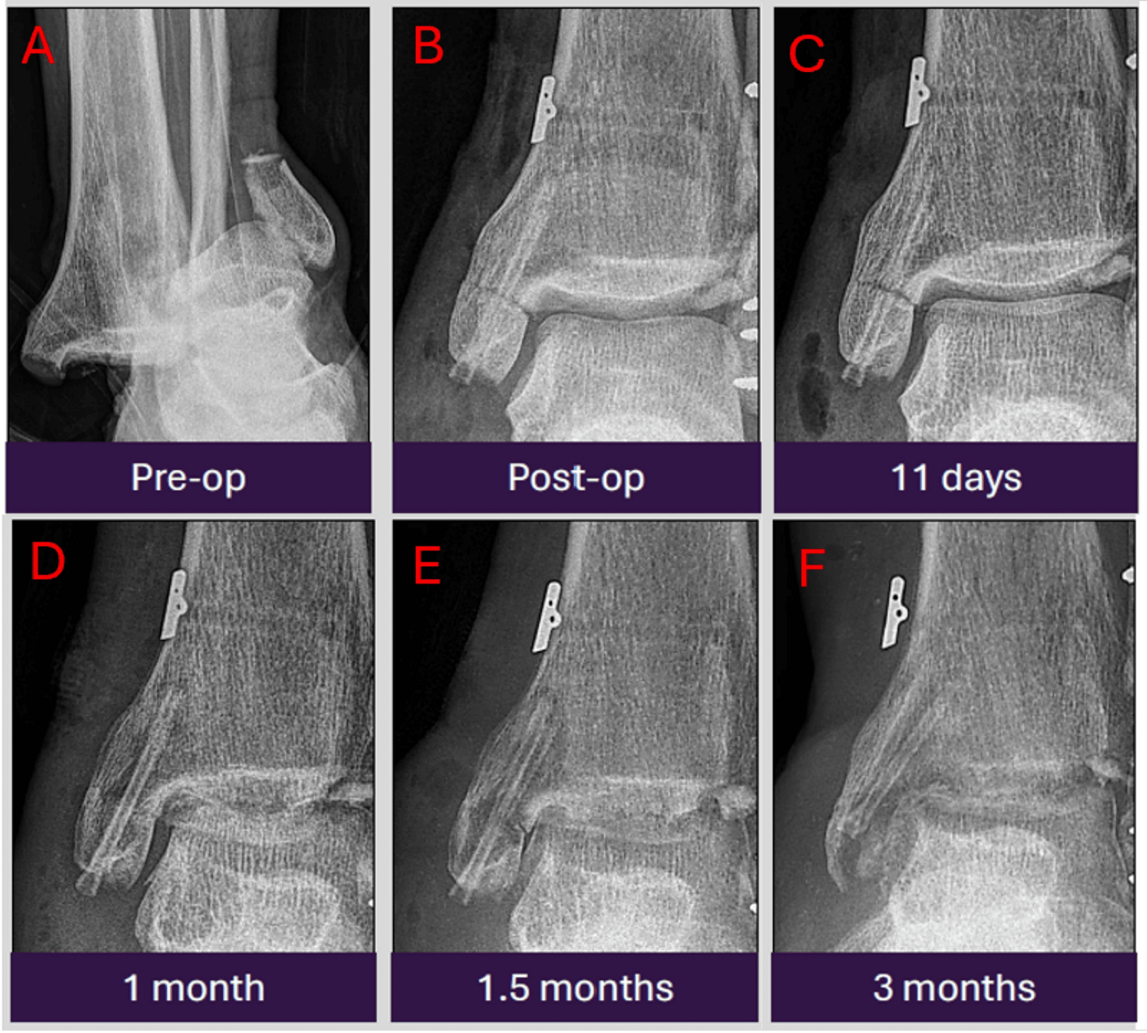
Serial antero-posterior radiographic images from pre-operation to three months post-operation showing peri-implant radiolucencies which are considered a normal feature of biocorrosion in the absence of clinical deterioration. The radiographs were obtained at pre-operation (A), immediately post-operation (B), 11 days (C), one month (D), 1.5 months (E), and three months (F) post-operation.

This case presents a 54-year-old chronic smoker with a trimalleolar ankle fracture who underwent surgical fixation using a Zimmer fibular plate, MAGNEZIX screws, and ZipTight device, along with bone grafting. After a month, the patient developed bogginess and tenderness of the wound with fever and elevated inflammatory markers. The patient underwent surgical abscess incision and drainage. A month after this, there was recurrent swelling over the medial ankle, suspicious for osteomyelitis and an infected hematoma, prompting further debridement and joint washout. With antibiotics, dressings, and smoking cessation advice, the patient improved and remained well (Figures 7A-7F). This case highlights how persistent and evolving peri-implant lucencies, associated with bone resorption and soft-tissue swelling when paired with clinical deterioration, may signal pathological complications, such as infection, rather than the expected magnesium implant degradation. This emphasizes the need to correlate radiographic changes with clinical context.

Case 7: pathological peri-implant lucencies with hardware loosening and infection

**Figure 8:**
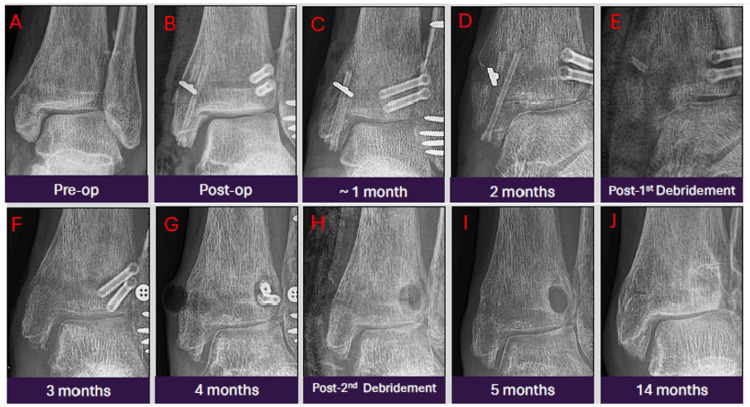
Serial antero-posterior radiographic images from pre-operation to 14 months post-operation showing pathological changes, such as screw extrusion, breakage and infective changes. The radiographs were taken at pre-operation (A), immediately post-operation (B), one month post-operation (C), two months post-operation (D), post-first debridement (E), three months post-operation (F), four months post-operation (G), post-second debridement (H), five months post-operation (I), and 14 months post-operation (J).

This 63-year-old patient with glucose intolerance underwent open reduction and internal fixation for a trimalleolar ankle fracture. By the first post-operative month, mild fever, wound dehiscence, and swelling developed. Antibiotics were initiated. Though there was no pus, over-granulation tissue was noted. By the second month, a screw spontaneously extruded with associated discharge. Radiographs showed increasing peri-implant lucencies, bone resorption, altered orientation of the endobutton, and a broken medial malleolar magnesium screw, all concerning for pathological changes. The next day, the patient underwent wound debridement and was restarted on antibiotics. The wound appeared to improve, but by the fourth month, the patient re-presented with discharge and pus from the lateral aspect of the wound. Radiographs showed increased peri-implant lucencies and a cloaca, highlighting chronic bony infection. Soft-tissue gas was also seen at the medial malleolus with increased bone resorption, suggesting infection. Repeat surgical debridement and complete implant removal were performed as the fracture had already united. Subsequent follow-up reviews showed improved ambulation with some residual stiffness (Figures 8A-8J). This case underscores the importance of distinguishing expected peri-implant gas from progressive lucencies and structural changes, which, in the presence of infection and clinical deterioration, signal true hardware failure and necessitate surgical intervention.

## Discussion

Self-dissolving materials, such as polymer-based and magnesium-based implants, have become increasingly important in recent years due to the implant-related disadvantages associated with metallic implants. In the field of foot and ankle surgery, polymer-based implants have been shown to have good functional outcomes [21,22]. An example is a prospective study by Kaukonen et al., which reported comparable outcomes between polyevolactic acid screws and metallic screws in syndesmosis fixation [23]. However, some disadvantages have been reported regarding polymer-based implants. Foreign body reaction is an associated issue [6,7]. In a study by Sun et al., it was reported that although there was comparable functional recovery between metallic and polyevolactic acid screws in the fixation of syndesmosis injuries, a higher incidence of foreign body reactions was observed in the polyevolactic acid screw group [24].

Biodegradable magnesium-based implants, such as MAGNEZIX CS screws, have been used with good effect in the field of orthopedic surgery, particularly for foot and ankle conditions [11-17]. For instance, several comparative studies between magnesium-based and titanium implants in the treatment of hallux valgus have shown comparable functional outcomes between the groups in terms of improvements in American Orthopedic Foot and Ankle Society (AOFAS) scores, Foot and Ankle Outcome Instrument (FAOI), and visual analog scores (VAS) [14-16].

Despite the advantages of MAGNEZIX screws, implant biodegradation introduces unique imaging challenges that can be misinterpreted as complications. This study highlights the importance of understanding and recognizing normal evolution and degradation patterns of magnesium implants. It has been shown that magnesium-based implants are expected to produce gas formation during degradation. Gas bubbles can result in tissue layer separation and subcutaneous irritation, but usually disappear within weeks after the initial surgery. Temporary surrounding radiolucency and soft-tissue reactions are expected but have no consequence [14,25]. Complete resorption of the implant has been demonstrated to occur after 12 months, accompanied by normal bone architecture, in a previous model study [8]. Implants can be expected to maintain full stability for up to 12 weeks [18].

Key principles in assessing radiographic imaging

Peri-implant gas, radiolucencies, and cortical remodeling are expected and are not pathological in the absence of clinical symptoms. It is essential to correlate radiographic findings with clinical symptoms, such as pain, fever, or elevated inflammatory markers, to prevent overinterpreting benign imaging findings. Serial radiographs are useful in monitoring for implant evolution and minimizing unnecessary advanced imaging, such as CT or MRI scans. In addition, effective communication between radiologists and surgeons is paramount to avoid misinterpretation and a disconnect between radiographic and clinical findings. Lastly, staying up-to-date with the latest literature on magnesium-based implants is crucial for refining interpretation and enhancing patient outcomes. Table 2 summarizes common pitfalls during the assessment of radiographic findings of magnesium-based implants.

**Table 2:** Common pitfalls in interpreting magnesium-based implant radiographs.

Expected imaging findings	Rationale behind findings	Mistaken for/pitfall	Red flag clinical findings
Peri-implant radiolucent zones seen in early post-operative imaging	Hydrogen gas formation	Osteomyelitis or implant loosening	Pain, fever, swelling, and raised inflammatory markers
Progressive decrease in implant density	Implant degradation and size reduction	Hardware failure or osteolysis fragmentation	
Fragmentation or nonuniform degradation	Irregular margins and inhomogeneous density	Implant fracture	
Periosteal reaction, callus formation, and bone regeneration	Cortical remodeling and new bone formation	Stress fracture or infection	
Soft tissue gas, soft tissue swelling, or mild joint effusion post-operatively	Soft tissue changes	Septic arthritis	

Limitations

This study has several limitations. Firstly, no patient-reported outcomes or statistical analyses were performed, as this is a descriptive paper. Secondly, there is a small population size containing only patients who underwent MAGNEZIX screws fixation and no other fixation group for comparison purposes. Further research on the clinical outcomes of magnesium-based implants, along with a direct comparison with metallic and other biodegradable implants, would be beneficial to the existing literature.

## Conclusions

Biodegradable implants, such as MAGNEZIX screws, are an innovative and effective alternative to metallic implants in orthopedic surgery. However, knowledge of the expected imaging findings of magnesium-based implants after insertion is essential in reducing unnecessary advanced imaging and misdiagnosis. Several imaging findings may be misinterpreted as complications; however, the most frequent pitfall when assessing radiological images of magnesium-based implants is confusing the expected radiolucencies from hydrogen gas generated during implant degradation with signs of infection or loosening. This article seeks to showcase specific cases that demonstrate critical radiological features of magnesium-based implants, equipping surgeons and radiologists with the expertise to differentiate expected imaging findings from those indicative of complications. Key principles in interpreting radiological findings of magnesium-based screws include (1) knowledge of expected peri-implant gas and radiolucencies on radiographs, (2) correlating radiographic findings with clinical assessment, (3) serial radiographic assessment, and (4) effective communication between radiologists and surgeons.
